# Against All Odds, Femicide Did Not Increase During the First Year of the COVID-19 Pandemic: Evidence From Six Spanish-Speaking Countries

**DOI:** 10.1177/10439862211054237

**Published:** 2021-11-26

**Authors:** Marcelo F. Aebi, Lorena Molnar, Francisca Baquerizas

**Affiliations:** 1 University of Lausanne, Switzerland; 2 University Pompeu Fabra, Barcelona, Spain

**Keywords:** femicide, routine activities theory, COVID-19 pandemic, lockdowns

## Abstract

This paper tests a *situational hypothesis* which postulates that the number of femicides should increase as an unintended consequence of the COVID-19-related lockdowns. The monthly data on femicides from 2017 to 2020 collected in six Spanish-speaking countries—Argentina, Chile, Paraguay, Panama, Mexico, and Spain—and analyzed using threshold models indicate that the hypothesis must be rejected. The total number of femicides in 2020 was similar to that recorded during each of the three previous years, and femicides did not peak during the months of the strictest lockdowns. In fact, their monthly distribution in 2020 did not differ from the seasonal distribution of femicides in any former year. The discussion criticizes the current state of research on femicide and its inability to inspire effective criminal polices. It also proposes three lines of intervention. The latter are based on a holistic approach that places femicide in the context of crimes against persons, incorporates biology and neuroscience approaches, and expands the current cultural explanations of femicide.

## Introduction

On March 28, 2020, roughly 2 weeks after the beginning of the stay-at-home restrictions imposed across the world to reduce the spread of the COVID-19 pandemic, *The Guardian* reported that “Lockdowns around the world bring rise in domestic violence,” with a subtitle that “Activists say pattern of increasing abuse is repeated in countries from Brazil to Germany” (Graham-Harrison et al., 2020).^1^ Several days later, on April 12, 2020, an editorial in the *Journal of Clinical Nursing* raised similar arguments (Bradbury-Jones & Isham, 2020). Citing as examples, one domestic homicide recorded in Spain within 5 days of the lockdown’s implementation and “. . . an increased number of domestic homicides in the UK since the lockdown restrictions were enacted,” the editorial warned that “The emerging homicide numbers underline the serious and potentially devastating unintended consequences of the pandemic for victim-survivors of abuse” (Bradbury-Jones & Isham, 2020, p. 2048). The reasoning behind this worldwide concern is relatively clear: the convergence in a reduced space and for an extended period of time of potential victims and offenders, coupled with the absence of formal social control, should lead to an increase in domestic violence offenses. Probably because this hypothesis—which we will refer to as *the situational hypothesis* because it is based on the relevance of the situation in which a crime occurs rather than the offender’s motivation—is grounded in common sense, it was supported by experts from different fields and recounted in the most prestigious newspapers, usually accompanied with anecdotical evidence similar to that quoted above.

In criminology, Cohen and Felson’s (1979) *Routine activities approach* have formalized this line of reasoning. This theory has been the object of several critics (for a summary, see McLaughlin, 2019), but in the context of the pandemic, we were unable to find any trace of them in the public discourse, nor did we find evidence that constructionists or postmodernists theorists were reassuring the potential lock-downed victims by telling them that “crime does not exist” (Christie, 2004; Hulsman, 1986). Sometimes, reality strikes hard.

The relevance of this apparent consensus about the conditions under which domestic violence increases must not be underestimated, as it can provide the support needed to introduce amendments to the criminal law and the criminal policies applied to prevent that crime. The question is whether the empirical evidence corroborates the reasoning behind that consensus.^2^ In that context, someone could object, as one anonymous reviewer of this paper did, that the length of the exposure to the risk of becoming a victim—increased by the fact that the lockdowns forced intimate partners to spend more time together—does not necessarily play a role in the theoretical framework of the routine activities approach. If that was the case, then the lockdowns would not lead to an increase in domestic aggression.^3^ We will keep that possibility as an alternative hypothesis, although we have found no traces of it in the literature on the COVID-19 pandemic’s effect on crime.^4^ In the meantime, the primary research question of this article is whether the data collected during the first year of the pandemic supports this situational hypothesis or not. We intend to answer that question by focusing on the most extreme form of violence against women and using data from six countries that are treated seldom in the international criminological literature. The reasons for these choices are explained in the following sections.

## An Empirical Contribution to the Southernization of Criminology

It has become relatively common to criticize the fact that criminologists focus their research on the so-called *Global North* (see, e.g., Carrington et al., 2018). Without entering into a conjectural debate about the reasons for that state of affairs, we consider it obvious that there is a lack in empirical research in the so-called Global South and that it is necessary to begin to fill that gap.

From that perspective, the sample of countries used in this article was drawn first from Latin American nations. It includes five countries—Argentina, Chile, Mexico, Panama, and Paraguay—that have introduced specific legislation on homicides against women and have published monthly statistics on them since at least 2017, which provides a reasonable framework for trend comparisons (see the Data Analysis section). These are all Spanish-speaking countries, which simplifies the comparison with Spain, a European country that also meets the requisites above.

With respect to COVID-19’s effect, the six countries studied are no exception to the deterioration in the quality of life that the pandemic generated around the world.^5^ By mid-March 2020, all of them imposed mandatory lockdowns to control the virus’s spread (see Table 1). On the basis of the data available, the limitations of which are known widely (Morris & Reuben, 2020), one can say that Spain, Panama, and Mexico appear to have been affected the most with respect to deaths during 2020, while the figures remained relatively low in Paraguay.

**Table 1.** COVID-19 Related Indicators in 2020 in Six Countries.

Country	Infected	Deaths	Population	Proportion infected	Proportion of deaths	Lockdown
Argentina	1,640,718	43,245	44,490,000	3.69	0.10	20 March
Chile	618,191	16,608	18,730,000	3.30	0.09	18 March
Mexico	1,413,935	124,897	126,200,000	1.12	0.10	23 March
Panama	253,736	4,022	4,177,000	6.07	0.10	25 March
Paraguay	109,073	2,262	6,956,000	1.57	0.03	20 March
Spain	2,009,975	58,827	46,940,000	4.28	0.13	15 March

*Source*. Worldometers.info (n.d.).

## Femicide, Feminicide, Domestic Homicide, Intimate Partner Homicide, or Female Homicide?

All of the countries under study collect data on femicide, but none defines it in precisely the same way. This comes as no surprise, as a similar diversity characterizes the scientific literature on this crime. In practice, the increasing number of studies on violence against women has led to an increase not only in the terms used to refer to the murder of a woman, but also in the definitions of these terms. In the case of *femicide*, these range from etymological interpretations—all murders in which the victim is a woman—to definitions that require that a current or recent male partner is the perpetrator, continuing through different kinds of combinations of the relationship between the perpetrator and the victim. In turn, this diversity influences the comparability of the data collected to measure that form of murder, and therefore, this article does not include any cross-national comparison of femicide rates.

Table 2 presents the legal definitions applied in each country, together with information on the sanctions foreseen compared to those applied for simple homicides. Table 2 also includes the total number of homicides against women and the number of femicides in 2018, the latest year for which both indicators were available. The reader is asked to keep in mind that this is not a comparative criminal law article, which implies that we will not enter into each definition’s legal subtleties. The goal is to illustrate the main similarities and differences across definitions. It is also worthwhile to mention that from an abstract point of view, legal definitions do not necessarily coincide with the operational definitions used when the data are collected. However, in this concrete study, only the empirical data from Spain correspond to a definition narrower than the legal one (see below).

**Table 2.** Legal Definitions of Femicide, Sanctions for Femicide and Simple Homicide, Number (and Rate Per 100,000 Population) of Women Victims of Homicide, and Number of Femicides in Six Countries.

Country	Law^a^	Sanction^a^	Definition^a^	Sanction for simple homicide (imprisonment for . . .)^a^	Number (and rate per 100,000 inhabitants) of women victims of homicide in 2018^b^	Femicides in 2018^c^
Argentina	Art. 80 CC and art. 4 Law 26 485	Life imprisonment	The victim of the homicide is a woman, the perpetrator is a man, and there is “gender violence”. The latter implies acts or omissions based on an “unequal relationship of power” that affect a woman in any way.	8–25 years	391 (0.88 per 100,000 inhabitants)	281
Chile	Art. 390bis CC	Imprisonment from 17 to 20 years or life imprisonment	*Femicide* (narrow definition): The victim is a woman and (1) she is or has been the spouse or partner of the perpetrator or (2) she has not cohabited with the perpetrator, but is having or has had a romantic or sexual relationship with him.	10–15 years	94 (0.5 per 100,000 inhabitants)	41
Mexico	Art. 325 CC	40–60 years of imprisonment, as well as 500–1,000 day fines	*Femicide* (extended definition, spelled as *feminicide*): The victim is a woman and the perpetrator (man or woman) has killed her for “gender reasons”. The latter occur when there was a sentimental, affective, or trusting relationship with the perpetrator, or the victim was physically or sexually aggressed, or she had been threatened, mistreated, or aggressed before by the perpetrator (at home, at work, or at school), or her corpse was publicly exposed.	12–24 years	3,769 (2.99 per 100,000 inhabitants)	893
Panama	Art. 132-A CC	25–30 years of imprisonment	*Femicide* (extended definition): The victim is a woman and the perpetrator (man or woman) is the partner, a rejected partner, a relative, or there is a relationship of subordination, or a physical or psychological vulnerability, or the victim is pregnant, or is killed by her condition of being a woman, or in front of her sons or daughters, or by vengeance, or using rituals, or there is a link with a sexual aggression or disrespect of the corpse.	10–20 years	37 (0.89 per 100,000 inhabitants)	20
Paraguay	Art. 50 Law 5 777	10–30 years of imprisonment	*Femicide* (extended definition, spelled as *feminicide*): The victim is a woman and the perpetrator (man or woman) has killed her (a) because of her condition as a woman and (b) is a current or previous partner, or a relative, or has been rejected as a romantic partner, or there has been a “cycle of violence” or a sexual aggression before, or there is a relationship of subordination, or a physical or psychological vulnerability.	5–15 years	63 (0.91 per 100,000 inhabitants)	57
*Spain*	Arts 138, 23 and 22.4 CC	Imprisonment from 12 to 15 years	Homicide aggravated by a close relationship or by “gender reasons”. The latter refer to “gender-based violence against women” as defined in art. 3.d of the Istanbul Convention.	10–15 years	117 (0.25 per 100,000 inhabitants)	51

*Note.* CC = criminal codes.

a *Source.* CC of each country. ^b^*Source.* https://datosmacro.expansion.com/demografia/homicidios (with references). ^c^Sources in Data and Methods section.

Table 2 shows that Argentina and Spain do not include femicide as a specific offense in their criminal codes (CC), although they foresee an aggravated punishment for the man who kills a woman for “gender-based violence” or “gender-based reasons,” respectively. The remainder of the countries include such an offense, denoted either as femicide (*femicidio*, in Chile and Panama) or feminicide (*feminicidio*, in Mexico and Paraguay). Independent of the label, the narrowest definition is that of Chile, which corresponds roughly to what researchers define as *intimate partner homicide* (IPH) and requires the perpetrator to be a man who is or has been his victim’s husband, companion, or romantic or sexual partner. In Spain, the data collected are based on an operation definition of femicide that corresponds to IPH, although its legal definition remains much broader as it combines three articles of the CC and art. 3.d of the *Council of Europe Convention on preventing and combating violence against women and domestic violence* (known as the Istanbul Convention, 2011), which states that “Gender-based violence against women’ shall mean violence that is directed against a woman because she is a woman or that affects women disproportionately.” The notion of killing a woman because she is a woman—whose operationalization remains a mystery—also appears in Panama and Paraguay’s codes. These two countries, together with Argentina and Mexico, use broad definitions of femicide. In Argentina, for example, “gender violence” is defined in a specific law on violence against women as any conduct, action, or omission, in the private or public spheres, on the part of individuals, the State, or its agents, which is based on an unequal relationship of power that affects the life, freedom, dignity, integrity (either physical, psychological, sexual, economic, or patrimonial), or the personal safety of a woman (art 4. Law 26 485). The notion of a relationship of subordination also appears as one of the reasons that qualify the act as a femicide in the CC of Panama and Paraguay, which also consider the killings relatives commit as femicides. Furthermore, in Mexico, the existence of a relationship of affection or trust is sufficient to consider the killing a femicide. The last three countries do not require the perpetrator to be a man, although in practice, femicides perpetrated by women have not attracted researchers’ attention and appear to be extremely difficult to prove, particularly if one has to establish that a woman was victimized by another woman because of her sex or gender.

In any case, Table 2 shows that even the broadest definitions of homicide do not include all cases in which a woman is killed. For example, in Mexico, in 2018, there were 893 femicides among a total of 3,769 women victims of homicide. The latter corresponds to a rate of roughly three women killed per 100,000 inhabitants, which is the highest observed in the sample of countries;^6^ logically, femicides are not presented as rates because of the differences in the definitions. It can also be seen in Table 2 that there are major disparities in the prison sentences for femicide. The latter range from up to 15 years in Spain, 20 in Chile, 30 in Panama and Paraguay, 60 in Mexico, to life imprisonment in Argentina.

Finally, one common feature of all of the definitions is that whenever they refer to a former relationship, they do not require the latter to have ended within a limited previous timeframe. Hence, a literal interpretation of these definitions leads to the conclusion that, in the eyes of the law, each relationship ties the persons involved during their lifetime.

## Previous Research: The COVID-19 Pandemic as a Natural Experiment

Empirical criminologists perceived the introduction of the lockdowns as the initiation of a natural experiment—“the largest criminological experiment in history” according to Stickle and Felson (2020)—and they focused on their effects on crime trends immediately. The first research results suggested that there was a drop in the bulk of crime that produced an immediate decrease in the European prison population rates (Aebi & Tiago, 2020), although that trend differed according to the type of offense. In particular, property crimes decreased (Halford et al., 2020; Hodgkinson & Andresen, 2020), but there was an increase in commercial burglaries (Hodgkinson & Andresen, 2020), hate crimes against East Asians and care providers (Eisner & Nivette, 2020), and in cybercrime (Buil-Gil et al., 2020). In their global analysis of trends in 27 cities in 23 countries in America, Europe, the Middle East, and Asia, Nivette et al. (2021) found that the lockdowns were related to a 37% drop in urban crime overall. These authors used the stringency index Hale et al. (2021) developed to measure the intensity of the lockdowns and found a negative correlation between the latter and the extent of the drop in urban crime: the tighter the lockdown, the greater the decline in crime. They did not observe a displacement to other offline crimes, but did not have a measure of trends in online crimes (Nivette et al., 2021).

As expected, the leading theoretical framework these studies employed was the *routine activities approach* (Cohen & Felson, 1979; Felson, 1995) mentioned above, which considers that crime is the result of the confluence in time and space of a motivated offender and a suitable target in the absence of capable guardians. A lockdown means that people spend less time in the streets and more time at home and in cyberspace; consequently:

The following predictions can be made: Personal victimisations in the public sphere (such as the ones resulting from fights, robberies and thefts in the streets) should decrease, while those in the private sphere (resulting from domestic violence offences) and on the Internet (cybercrimes) should increase. (Aebi & Tiago, 2020, p. 3)

For instance, from that perspective, the lack of guardianship that rendered the premises vulnerable explains the increase in commercial burglaries (Hodgkinson & Andresen, 2020), while the drop in high-volume crimes suggests that the decline in urban mobility reduced the opportunities and increased guardianship in households (Nivette et al., 2021).

Against that background, intimate partner violence (IPV) during the lockdowns received extensive attention from researchers and was even qualified as “a pandemic within a pandemic” (Evans et al., 2020). In general, studies around the world were consistent in finding a moderate increase in the global number of agressions between intimate partners at the time of the lockdowns and, more broadly, throughout the first year of the pandemic (Arenas-Arroyo et al., 2021; Campbell, 2020; Eisner & Nivette, 2020; Evans et al., 2020; Gosangi et al., 2020; Mohler et al., 2020; Piquero et al., 2021). For example, Arenas-Arroyo et al. (2021) studied trends in IPV in Spain through an online survey posted on social media (*N* = 13,786 adult women, not strictly representative) and observed an increase in psychological violence, but no evidence of a rise in physical violence. Piquero et al. (2021) conducted a systematic review that corroborated this moderate increase in IPV, which they interpreted in relation to the increased strain provoked by the pandemic’s collateral effects on financial instability, home schooling, and illness or deaths as a consequence of the coronavirus, as well as the mental health problems provoked by the social distancing impositions. Correspondingly, and despite the difficulties of calling the police while confined with the aggressor, there was a rise in domestic violence-related phone calls since the beginning of the pandemic in several countries, including the United States (Bullinger et al., 2020) and Argentina, where the hotline for domestic violence prevention recorded increases between 16% and 27% during each month from April to October 2020.^7^ In that context, one could also argue that the similarly confined neighbors may have acted as *capable guardians*, intervening directly or calling the police in case of aggression, and therefore contributing to the increase of the known cases of IPV during the lockdowns.

Finally, from the beginning of the stay-at-home restrictions, several scholars assumed that femicide would follow the same trend as IPV (Boman & Gallupe, 2020; Kofman & Garfin, 2020; Lund et al., 2020; Weil, 2020).^8^ This assumption appears logical as, despite the differences across countries mentioned above, all definitions of femicide include IPH, which constitutes the most extreme form of IPV. In addition, several research reviews—based largely on studies conducted in the United States—nurture the hypothesis of a crescendo from nonlethal to lethal domestic violence, which has inspired many laws around the world and constitute the basis of the predictive tests developed since the 1990s without much success. For example, according to Campbell et al.’s (2007) literature review, “the major risk factor for intimate partner homicide (IPH), no matter if a female or male partner is killed, is prior domestic violence” (p. 246). Spencer and Stith’s (2020) meta-analysis also identified several previous types of domestic violence (*e.g*., threats, nonfatal strangulation, forced sex, or stalking) as well as substance abuse (which includes both drug and alcohol abuse) as risk factors for IPH.

In contrast, the results of a minority of studies have suggested that the perpetrator of a femicide does not have a specific profile but is more like an “ordinary guy” (Dobash et al., 2004), a concept that resonates with that of Hannah Arendt’s (1963/2006) *banality of evil*. These studies (for a review, see Schaller, 2021) have insisted, at least since the early 1990s, that intimate partners’ murders are triggered often by the victim’s decision to end the relationship (see *e.g*., Cusson & Boisvert, 1994). That would explain why a substantial number of the femicides are committed by previous partners who often have no previous arrest record.^9^ In principle, this type of femicide should not increase during a lockdown because former partners’ movements are restricted and because the lockdown reduces the chance to end the relationship with a current partner and move to another place.

Within that framework, the first research results from three different countries did not support the hypothesis of an increase in femicides during the first year of the pandemic. For example, Hoehn-Velasco et al. (2021) observed that femicides in Mexico remained stable during the lockdown and even declined in some municipalities; moreover, they found a negative correlation between men’s unemployment and femicides; however, they did not provide a specific explanation for this paradoxical finding. In Peru, Calderon-Anyosa and Kaufman (2021) studied homicide trends from 2017 to 2020 and found that the total number of women victims of homicide declined during the lockdown. They attributed this decline to the increase in the number of police officers patrolling the streets and to the difficulties that perpetrators would have disposing of the corpse. In Turkey, Asik and Nas Ozen (2021) compared trends in IPH during 2020 with those from 2014 to 2019 and found that IPH decreased considerably during the first year of the pandemic. They attributed the decrease to the curfew that accompanied the lockdown, which prevented ex-partners from reaching their victims.

## Data and Methods

### Data on Femicides

Data on the monthly number of femicides were collected from reports published by official bodies as well as by organizations that lobby for women’s rights in each country. The sources are as follows:

- Argentina. Observatory of Femicides (*Observatorio de Femicidios del* Defensor del Pueblo de la Nación, 2018, 2019, 2020, 2021).
- Chile. *Annual Report on Femicide of the Intersectoral Circuit*, published by the *Ministerio de la Mujer y la Equidad de Género* [Ministry of Women and Gender Equity (Chile) (2018, 2019, 2021)].
- Mexico. Website of the Government of Mexico (Secretaría de Seguridad y Protección Ciudadana, 2021).
- Panama. Reports of the Attorney General’s Office of the Public Ministry of Panama (*Procuraduría de la Nación del Ministerio Público de Panamá*) (2018, 2019, 2020).
- Paraguay. Observatory for Women, dependent from the Ministry of Women of Paraguay (Ministerio de la Mujer de Paraguay, 2018, 2019, 2020a, 2020b).
- Spain. Europa Press (2020).

### The Stringency Index

To measure the length and intensity of the lockdowns worldwide, Hale et al. (2021) developed the *stringency index*, which “. . . refers to the containment and closure policies, sometimes referred to as lockdown policies” (Hale et al., 2021, p. 536). The index provides a daily measure of the lockdowns’ intensity in each country. The higher the index, the tighter the lockdown. For this article, we have computed each country’s monthly average stringency index.

### Control Variable: The Seasonal Distribution of Femicide

Following the publication of the first comprehensive crime statistics in France, Quételet (1833) observed an increase in property offenses in winter coupled with an increase in offenses against persons in the summer. He attributed the latter to the proliferation of people in public spaces, but also to the effects of climate variations on human behavior. The second explanation was later discarded as nonscientific, but the first is a pillar of the routine activities approach (Cohen & Felson, 1979). Hence, as this article compared the number of femicides month by month during several years, it is imperative to use the seasonal distribution of that crime as a control variable.

**Figure 1.**
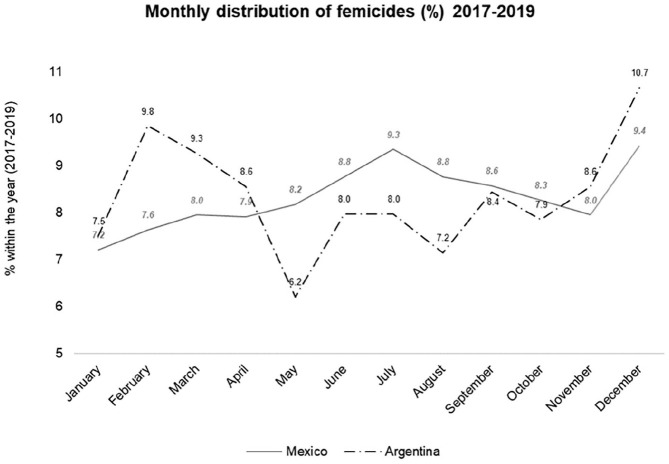
Monthly percentage distribution of the femicides committed in the years 2017 to 2019.

**Figure 2.**
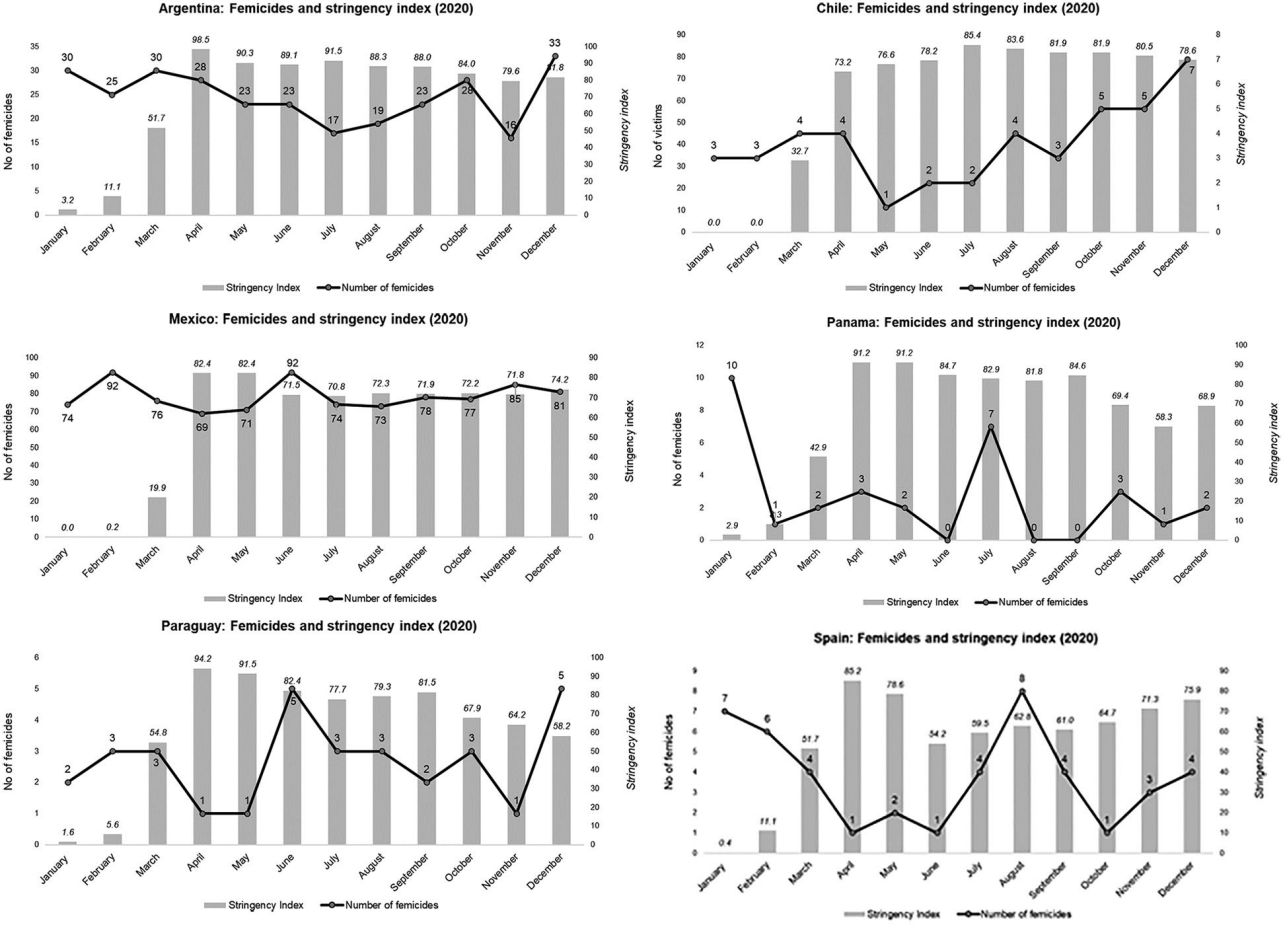
Femicides in 2020 and stringency index (2020).

From that perspective, Figure 1 presents the monthly distribution of femicides in Argentina and Mexico from 2017 to 2019. To obtain a sufficient number of observations, we first added the number of femicides in each month of the three years and then presented their distribution in percentages by month. The choice of the countries is attributable not only to the fact that Argentina is in the Southern Hemisphere and Mexico in the Northern, but also to the fact that, even when the three years are added, none of the other countries reached at least a minimum of ten observations by month, which is usually the minimum number of observations per variable required to conduct reliable regression analyses (Altman, 1990). Overall, in Argentina, the greatest numbers of femicide victims during the years 2017 to 2019 were recorded during the months of December and February. In Mexico, the peaks were recorded in December and July. The common point is December, which in countries with a Christian tradition corresponds to the Christmas season. Compared to other periods of the year, during that season, there are more people in the streets buying presents, there are more meetings of friends and colleagues celebrating the end of the year, and there are more family reunions to celebrate Christmas and the New Year. The difference between both countries is that the second peak takes place in February in Argentina and in July in Mexico, thus coinciding with the summer in the Southern and Northern Hemisphere, respectively. This indicates that the seasonal distribution of femicides coincides partially with the general distribution of crimes against persons which, according to contemporary research around the world, continues to peak during the summer (Carbone-López, 2017). One possible explanation is that both countries use broad definitions of femicide. Nevertheless, even in countries where the definition is narrow and corresponds to IPH, such as in Spain, it has been observed repeatedly—and can be seen in Table 3 and Figure 2—that there are peaks in the summer and near the end of the year holidays, which have been attributed traditionally to the fact that those are seasons in which families spend more time together (Cerezo-Domínguez, 2000). That explanation is also inspired by the routine activities approach (Cohen & Felson, 1979), which entails some overlap—crimes against persons increase because there are more people in the streets during the hot season, while femicides increase because families spend more time together during the summer holidays—and may hide subtler interactions, such as those between former partners.

**Table 3.** Monthly and Annual Number of Femicide Victims From 2017 to 2020 in Six Countries, and *Z*-Score, Weighted Average, and Standard Deviation in Each Country.

Country	Year	January	February	March	April	May	June	July	August	September	October	November	December	Total	*Z*-score	Weighted average	Weighted *SD*
Argentina	2017	21	35	25	30	18	22	31	15	21	19	17	38	292	1.73	282.3	7.3
	2018	19	27	24	26	16	25	20	19	27	25	33	20	281			
	2019	24	22	30	17	19	21	17	27	24	23	23	33	280			
	2020	30	25	30	28	23	23	17	19	23	28	16	33	295			
**Chile**	2017	3	5	4	4	6	3	1	4	3	4	5	3	45	−1.72	44.2	0.7
	2018	5	1	0	1	5	7	4	4	2	2	5	5	41			
	2019	5	4	2	4	3	6	2	5	3	3	4	5	46			
	2020	3	3	4	4	1	2	2	4	3	5	5	7	43			
Paraguay	2017	10	5	2	2	3	3	6	4	3	4	7	7	56	−1.73	48.3	9.4
	2018	5	6	1	4	6	2	1	6	6	7	9	4	57			
	2019	5	1	7	3	2	1	0	3	4	2	5	7	40			
	2020	2	3	3	1	1	5	3	3	2	3	1	5	32			
Panama	2017	3	2	2	0	1	2	0	1	3	0	2	2	18	1.73	21.2	5.7
	2018	1	2	1	1	1	2	3	1	1	3	2	2	20			
	2019	2	3	2	1	3	1	2	. . .	2	3	2	2	23			
	2020	10	1	2	3	2	. . .	7	. . .	. . .	3	1	2	31			
Mexico	2017	49	64	61	61	69	74	70	68	56	59	57	54	742	1.73	892.8	28.4
	2018	68	66	68	76	63	76	84	65	76	85	68	98	893			
	2019	69	67	76	67	79	76	87	93	89	69	80	91	943			
	2020	74	92	76	69	71	92	74	73	78	77	85	81	942			
Spain	2017	5	10	3	4	6	3	2	4	2	5	3	3	50	−1.73	52.8	4.5
	2018	2	2	3	4	1	6	8	7	10	4	2	2	51			
	2019	8	3	4	5	3	7	10	3	4	4	3	1	55			
	2020	7	6	4	1	2	1	4	8	4	1	3	4	45			

The seasonal distribution observed in Figure 1 raises doubts about the pertinence of several criminological theories to explain crimes like femicide. For example, why would strain be greater or self-control lower during the summer? Or why would the labeling effect, the social learning process, or the consequences of a patriarchal society manifest themselves predominantly during that season? In addition, the consistency of the trends observed in Figure 1 illustrates the need to take into account the seasonal distribution of femicides when analyzing the effect of the stay-at-home restrictions on that kind of murder. In particular, as the lockdowns did not last the entire year, the increases or decreases observed could not be attributable to them alone but to the usual seasonal variations in femicide as well.

### Data Analysis

We use threshold models to measure whether the number of femicides recorded in each country in 2020 differed significantly from the average number recorded from 2017 to 2019. Threshold models in statistics were developed during the second half of the 20th century and, building on Chamberlayne’s previous work, Bruce (2008, 2012) proposed a modern approach to them in 2008 with a focus on the analysis of crime data. That approach was applied by Maldonado-Guzmán et al. (2020) for the study of trends in property crime in Spain, and it is the one used here.

In the first step, a threshold analysis estimates the expected number of crimes in a given year on the basis of the levels of crime observed in the previous years; in the second step, the analysis compares that expected volume of crime to the one observed in reality; finally, it uses *Z*-scores to measure the difference. *Z*-scores represent the number of standard deviations that separate the value observed from that expected. Bruce (2008, 2012) indicates that researchers can choose the standard value from which the threshold indicates an increase or decrease in crime, although it has become customary to consider that a *Z*-score between −1.5 and 1.5 reflects stability. Maldonado-Guzmán et al. (2020) recommend extending that range to −2 and 2, and we follow their recommendation here. Bruce (2008, 2012) advises working with annual data and including at least the three years preceding that under study. We follow both advises for our threshold analysis, and we also require monthly data on femicide for each of these years for our analyses of the intensity of the lockdowns based on the stringency index.

Concretely, we begin by computing the weighted moving average for 2017 to 2019 by weighting the number of femicides in 2017 by 1, those in 2018 by 2, and those in 2019 by 3. Then, we sum up these weighted values and divide them by the sum of the weights (in this case, by 6):

$$Weighted\;\bar{x}=\;\frac{\left(2017.1\right)+\left(2018.2\right)+\left(2019.3\right)}{6}$$

Thereafter, we compute the standard weighted deviation for 2020 compared to the period 2017 to 2019 (*x_i_* is the number of femicides in 2020 and *N* the number of years, in our case 3).

$$Weighted\;SD=\;\sqrt{\frac{{\left(x_{i}-Weighted\;\bar{x}\right)}^{2}}{N-1}}$$

Finally, we compute the weighted *Z*-score. The threshold technique bases its estimates upon this coefficient, which corresponds to the number of standard deviations above or below the weighted moving average for the previous years. To calculate the weighted *Z*-score, we subtract the weighted average of the number of femicides in 2020 from those committed from 2017 to 2019 and divide the product by the weighted standard deviation:

$$Weighted\;Z\text{-}score\;=\;\frac{X\;-\;Weighted\;\bar{x}}{Weighted\;\;SD}$$

## Findings

Table 3 shows the monthly number of femicide victims from 2017 to 2020 in the six countries under study. The table also presents the weighted average for the years 2017 to 2019, the weighted standard deviation for the year 2020 compared to that average, as well as the *Z*-score the comparison between 2020 and the period 2017 to 2019 yielded. Overall, the six *Z*-scores are below our threshold (±2), thereby indicating that, against all odds, the number of femicides remained stable during 2020 in comparison with the previous years.

In particular, Paraguay recorded 32 femicides in 2020, compared to a weighted average of 48 between 2017 and 2019 (*SD* = 7.3), which corresponds to a 33% decrease. A similar pattern was found in Spain, where the 45 femicides recorded in 2020 corresponded to a 15% decrease compared to the average 53 (*SD* = 4.5) during the previous years. Finally, Chile recorded 43 femicides in 2020, a number that is slightly lower (−2.3%) than the weighted average of 44 (*SD* = 0.7) femicides committed during the three previous years.

On the contrary, Argentina recorded 295 femicides in 2020 compared to a weighted average of 282 between 2017 and 2019 (*SD* = 7.3), which in percentage corresponds to a 4.6% increase. Mexico showed a similar pattern, in that the 942 femicides recorded in 2020 correspond to a 5.6% increase compared to the weighted average of 893 (*SD* = 28) for the three previous years. Finally, Panama recorded a weighted average of 21 femicides per year between 2017 and 2019 (*SD* = 5.6), but in 2020, the death toll was 32. Nevertheless, the distribution of femicides in 2020 is particularly skewed, as the country recorded a peak of 10 cases in January, when in previous years the number of victims during that month ranged between 1 and 3. That increase cannot be attributed to the lockdown, which was introduced in March. In fact, from February to December, the death toll was identical (21 victims) in 2020 and 2019.

This country-by-country analysis highlights the threshold analysis’s importance in estimating the stability or instability of the trends observed. The simple estimation of the percentage change in the number of femicides in 2020 compared to the weighted average for the years 2017 to 2019 produced several extreme values that without the threshold analysis, would have misled the interpretation. In summary, in three countries (Spain, Chile, and Paraguay) there were fewer femicides during 2020 than the mean number for the previous three years, while in the three others (Argentina, Panama, and Mexico) there were more, but the differences were not statistically significant. The distribution of the femicides observed in Panama also highlights the importance of a monthly analysis of their distribution that takes into account the tightness of the lockdowns while keeping the seasonal variation in mind. Figure 2 shows the monthly distribution of femicides compared to the stringency index.

Figure 2 shows that the stringency indices were at their highest in all countries in April and May 2020. This indicates that the lockdowns’ intensity was at their maximum during that time. However, in nearly all countries, these months coincide with those in which the number of femicides was at their lowest level. For example, there were between one and four homicides in Chile, Panama, Paraguay, and Spain during these months. In Mexico, where the definition of femicide is broader, April and May were also the months in which the monthly number of femicides was the lowest in the entire year. Finally, in Argentina, the number of femicides was decreasing during these months, thus following the seasonal distribution of femicides in the Southern Hemisphere, which decrease after the summer. Furthermore, the same trend can be observed in Chile and Paraguay, the other two countries in that hemisphere. Conversely, the peaks in the Northern Hemisphere—represented in this study by Mexico and Spain—also coincide with the seasonal distribution of femicide, which takes place in the summer and around the Christmas season. In summary, the trends in femicide in the six countries under study are unrelated to the intensity of the lockdowns.

## Discussion

### Contextualisation of the Findings

The main finding of our analyses is that, in Argentina, Chile, Mexico, Panama, Paraguay, and Spain, femicide neither increased during the first year of the coronavirus pandemic nor, in particular, during the months when the lockdowns were tighter. In fact, the monthly distribution of femicides in 2020 did not differ from their seasonal distribution in any given year, which peaks during the summer—January and February in the Southern Hemisphere, represented in this research by Argentina, Chile, and Paraguay; and July and August in the Northern Hemisphere, represented by Mexico and Spain^10^—and during the Christmas season, which in the Southern Hemisphere coincides with the beginning of the summer. This seasonal distribution of femicides was observed from 2017 to 2020 both in countries that use a narrow definition of femicide—Chile and Spain, where the definition corresponds to IPH—and in those that use a broader one—Paraguay, Panama, and Mexico. This indicates that the 2020 lockdowns did not lead to an increase in the number of women murdered by their cohabiting partners or relatives.

It is worth mentioning that the same pattern was observed in Colombia,^11^ a country that applies a very broad definition of femicide, but that could not be included in this research because monthly data on femicides are available for 2018, 2019, and 2020, but not for 2017, which was one of the conditions to be part of the sample. In addition, the absence of an increase of femicides during the entire 2020 year coincides with Hoehn-Velasco et al.’s (2021) observations in Mexico, Calderon-Anyosa and Kaufman’s (2021) in Peru—although in that case, the authors studied the more general category of women victims of homicide—Asik and Nas Ozen’s (2021) in Turkey, and the Federal Office of Statistics’ data in Switzerland (Office Fédéral de la Statistique [OFS], 2021).

These empirical results refute the situational hypothesis that, inspired by the routine activities approach (Cohen & Felson, 1979), postulated an increase in femicides during the lockdowns because of the confluence of a potential offender and a suitable victim in a reduced space and for an extended period of time in the absence of a capable guardian. In contrast, a similar situational hypothesis was corroborated with respect to nonlethal *domestic violence offenses* and IPV: the latter did increase during the first year of the pandemic, particularly during the lockdowns, according to research conducted in several countries around the world (Arenas-Arroyo et al., 2021; Campbell, 2020; Eisner & Nivette, 2020; Evans et al., 2020; Gosangi et al., 2020; Mohler et al., 2020; Piquero et al., 2021). In turn, this increase in nonlethal domestic violence and IPV refutes the alternative hypothesis presented in the introduction of this paper, which suggests that the time dimension—that is, the fact that the lockdown increased the amount of time a potential offender and suitable victim spent together in the absence of a capable guardian—should not be taken into consideration when testing hypotheses derived from routine activities theory.

The question then is why was the *situational hypothesis* refuted in the specific case of femicides but corroborated for nonlethal forms of domestic violence? One plausible explanation is that the dynamics of femicide differ from those of other forms of domestic violence and, given the broad definitions of femicide applied in some countries, that their relation with the dynamics of homicides in which kinship is not involved are quite complex. In particular, the definitions of femicide usually include two elements: (unbalanced) power and kinship. The latter implies some sort of affection, which should serve as a regulator of aggressive impulses. Nevertheless, affection does not play a major role in the criminological explanations of femicide. To put it bluntly, using a word seldom used in criminology papers, kinship implies *love*, which is the basic force that brings couples together and ties members of the same family. Naturally, love, which is extremely difficult to operationalize, is confused sometimes with attraction or infatuation, and can occasionally transform into hate, but one cannot ignore it when trying to explain murder between partners or relatives. However, mainstream femicide research ignores these real-world complexities often, which surely explains why criminology has not found a solid scientific explanation of femicide. This is particularly worrying if we expect policymakers to produce evidence-based criminal policies to protect women.

### Criminal Policy Implications

One factor that may have contributed to the state of affairs described in the previous paragraph is the proliferation of studies that seek to establish the profile of the murderers on the basis of known cases of femicides. This is a relatively inexpensive way to conduct research, as the researcher needs only to have access to the relevant documents—for example, the sentences the courts impose in femicide cases—but the methodological weaknesses of a research design that lacks a control group are known widely. In practice, this may explain in part why the literature reviews and meta-analyses presented in our section on previous research have concluded that prior domestic violence is the main predictor of IPH (see Campbell et al., 2007; Spencer & Stith, 2020).

What can a policymaker do with this kind of information? Let us take the concrete case of Spain where the police recorded, in round numbers, 70,000 offenses of domestic assault in 2017, 72,000 in 2018, and 77,000 in 2019, which led to the identification of 53,000, 55,000, and 59,000 suspected offenders, respectively (Ministerio del Interior [MIR], 2020, pp. 171 and 175). This is the equivalent of the total prison population of Spain, which on 31 December, 2019 was 58,517 inmates (MIR, 2020, p. 334). If, at the beginning of the pandemic, the experts foresaw an increase in IPH during the lockdowns and the best predictor of those is a previous history of domestic violence, should the policymaker order the preventive arrest of some of these suspected offenders? Now that our research has shown that Spain recorded one victim of IPH in April, two in May, and one in June 2020, it is clear that using previous arrests for domestic violence as a predictor of future IPH would result in an outrageous number of false positives, in the sense that 99.99% of the known domestic violent offenders did not become murderers. This corroborates the hypothesis that a crescendo from nonlethal to lethal domestic violence can have its origins only in retrospective studies based on the analysis of the previous records of known murderers and that, in practice, it has no ability to predict IPH properly. Researchers are familiar with this pattern, because it can be observed in many life activities. For example, nearly all hard drug addicts have consumed soft drugs before, but the vast majority of soft drug users do not become hard drugs addicts.

Can we criminologists blame policymakers for applying populist criminal policies or succumbing to ideology when we have not yet provided a valid scientific explanation that could inspire effective crime prevention programs? Babcock et al. (2004) showed the inefficiency of programs based on a feminist framework long ago, and recent meta-analyses corroborated that the “classic BIP [batterer intervention program] that relied solely on a feminist framework, a cognitive-behavioral model, or a mix of the two, is unlikely to provide a meaningful solution to the problem of intimate partner violence” (Wilson et al., 2021, p. 3). Nevertheless, evidence-based practitioners are likely to have a difficult time resisting the pressure of activists—often supported by government officials—to continue such programs as long as there are no realistic and efficient alternatives.

### Three Ways Forward

The complexity of the interactions between affection, power, opportunity, and gender highlights the need for a more holistic approach to studying and preventing femicides. We believe that there are three lines of research that, used in combination, can help reach that goal. First, it appears to us that it is time to place the study of femicide in a wider context. In that sense, femicide’s particular dynamics are more evident when studies are based on the analysis of all homicides recorded during one or more years. From that perspective, Wolfgang’s (1958) classic study, and that of Daly and Wilson (1982), based on all homicides recorded in Philadelphia from 1948 to 1952 and in Detroit in 1972, respectively, can shed some light on the stability of femicides that we observed from 2017 to 2020 in the countries that apply a broad definition of that crime. Both studies observed an over-representation of IPH—which was referred to as *spousal homicide* in those times—but a low proportion of genealogical relationships among all homicides: “The 6.3% of Detroit homicides that involved blood kin seems a remarkably low proportion in view of the likely frequency and intensity of social interactions” (Daly and Wilson, 1982, p. 372). These interactions were not referred to yet as a *situational factor*, but it can be seen that the researchers were surprised by their relatively weak effect, just as we are surprised today by the lockdowns’ lack of effect.

In that context, the missing element may well be a *punctual incident* that triggers the lethal assault, coupled with the availability of an instrument—a gun or, quite often in the examples Wolfgang (1958) provided, a butcher’s knife—capable of inflicting death. This suggests that our results fit relatively well the hypothesis—presented in the Previous Research section—that femicides are triggered frequently by a specific event which, in the case of IPH is often, according to Cusson and Boisvert (1994), the victim’s decision to end the relationship (see also Schaller, 2021). If we operationalize that hypothesis, the key element is not the victim’s decision in itself, but the fact that the perpetrator realizes that it is a *final* decision. This can happen because the perpetrator trusts the victim’s words or because he is confronted with empirical evidence of the fact that the relationship is over, for example, when he discovers that the victim has begun a new romantic relationship. The perpetrator can become aware of that fact either during the relationship or after a breakup, which explains the relatively high number of victims killed by previous partners. However, the decision to end a relationship and move out of a common house can seldom be taken during a lockdown, nor can a previous partner reach the potential victim who is living with a new partner already. This may explain in part why femicides did not increase during the lockdowns or, to put it differently, why the situational hypothesis is rejected by the data collected.^12^

From that perspective, the main criticism of the original version of the routine activities approach is the lack of definition of the *motivated offender* (Akers, 1999, pp. 30–31).^13^ Our results tend to corroborate the pertinence of that critique in the specific case of femicide, and we suggest that it is the awareness of the end of the relationship that may play a role in motivating the offender to take action.

As millions of relationships are broken—and new ones formed—every day around the world, the question becomes why does the vast majority of the former partners go on with their lives, but some aggress against, and even kill their partners? This led us to our second proposal with respect to lines of research. Now that it is clear to scientists that the nature-nurture debate is pointless because human behavior is the result of the combination of both (Pinker, 2002, 2011; Sapolsky, 2017), we consider that it is time to fully include biology and neurosciences as elements of criminologists’ basic training. In that respect, Raine (2014) pointed out that research on domestic violence is based almost exclusively upon a sociological perspective—which blames a patriarchal society that leads men to use power to control their feminine partners—while in fact the rare neuro-criminological studies in that field have shown that some batterers have a reactive aggressive personality, hence suggesting that there may be, at least in some cases, a neurobiological predisposition to battering. In our opinion, Sapolsky (2017) provided the most comprehensive and multidisciplinary view on the interaction between biology and the environment in his book *Behave*, which combines neurosciences, endocrinology, epigenetics, culture, evolutionary psychology, game theory, and comparative zoology. Sapolsky’s approach incorporates distant factors like the culture of origin or the levels of stress suffered during fetal life and early childhood, which vary widely across regions and could help explain the impressive differences observed in the rate of women killed across the countries studied in this paper (see Table 2). It also includes proximate factors, such as the levels of stress and trauma suffered during the weeks and months before the aggression, which can enlarge the amygdala, excite the neurons, lead the prefrontal cortex to atrophy, and thus facilitate a violent reaction (Sapolsky, 2017). This appears to be particularly relevant to the study of those femicides that take place following the deterioration or end of a relationship. Knowing that the amygdala plays a major role not only in violence, but in fear, can also help us understand what is going on in the mind of some aggressors when they are faced with an uncertain future. Even more important, the empirical research in which Sapolsky (2017) grounded his ideas shows that there is much room for change in a human brain during a lifetime. This indicates that there are major opportunities for intervention if the appropriate programs are developed, which is precisely the direction in which we believe criminology should move.^14^

Finally, Sapolsky (2017) is aware that cultures change throughout time, which leads us to Norbert Elias’s theory of the *civilizing process*,^15^ and our third proposal for lines of research. In fact, even for those who are reluctant or unprepared to introduce biology and neurosciences into criminology’s basic curricula, there is room for innovation within the purely sociological and cultural explanations of femicide. From that perspective, there are two elements in Elias’s (1939/2000) theory that deserve attention. First, he pointed out that during their lifetime, humans also go through a civilizing process, which makes them less and less aggressive. The work of Richard Tremblay with several colleagues has corroborated this remarkable intuition (for a summary, see Tremblay, 2008), showing that humans do not learn to become aggressive but, on the contrary, learn to act in a nonaggressive (*i.e*., in a *civilized*) manner. In fact, the levels of aggression shown in early childhood would be intolerable among adults. Again, this indicates that there is scope for improvement if we develop the appropriate programs. Second, as Linde and Aebi (2020) pointed out, Elias was a German Jew who was forced to seek exile in England before World War II, where he finished writing *The Civilizing Process* while Hitler was launching the genocide that took the lives of Elias’s parents in 1940 and 1941.^16^ Knowing this, it is possible to interpret Elias’ (1939/2000) purposes when he wrote the book in a different way. Yes, he was showing the way Western cultures reduced violence and became more *civilized* throughout the centuries, but at the same time, he was worried about how easily that civilizing process could be stopped and even reversed because of its fragility. This can be seen in his choice of words, that is, when he uses Freudian terminology to hypothesized that humans have “repressed” their aggressive predispositions.^17^ Elias (1939/2000) asks himself what it takes to awaken these predispositions and answers: “Immense social upheaval and urgency, heightened by carefully concerted propaganda, are needed to reawaken and legitimise in large masses of people the socially outlawed drives, the joy in killing and destruction that have been repressed from everyday civilised life” (p. 170). Similarly, knowing that humans also undergo a civilizing process, we should ask ourselves what it takes for some men to stop repressing their predisposition to aggress, and that is where the notion of becoming aware of the end of a relationship may be useful.

### Generalizability of the Findings

In the next and final section, we present our conclusions, but before that, we would like to emphasize that the limitations of the data available—which may suffer alterations in the months to come—and our limited sample size affect our results undoubtedly. Hence, our findings cannot be generalized, and we encourage researchers to replicate our study in other countries.^18^

## Conclusion

The goal of this paper was to test the *situational hypothesis*, which postulates that the number of femicides should increase as an unintended consequence of the lockdowns introduced to control the spread of the COVID-19 pandemic. The data collected in six Spanish-speaking countries—Argentina, Chile, Paraguay, Panama, Mexico, and Spain—led us to reject that hypothesis. In particular:

1. The total number of femicides in 2020 was similar to that recorded during each of the three previous years.
2. The number of femicides did not increase during the months of strict lockdown. Furthermore, in five of the six countries under study, the monthly numbers of femicides in April and/or May 2020 were the lowest in the entire year.
3. The distribution of femicides during 2020 followed the pattern of the seasonal distribution of femicides in previous years.
4. This pattern coincides partially with that of violent offenses, which peak during the summer, when there are more social interactions in the public sphere, but also when family members spend more time together.
5. The definitions of femicide differ considerably across the countries under study. They all include IPH, most include members of the same kin, and two are even broader.
6. Some countries define femicide as the act of killing a woman because of her gender/sex, but they do not specify the ways in which that reason to kill can be operationalized and proven in a court of justice; at the same time, most countries’ legislation discriminates against men on the basis of their gender/sex, in the sense that they apply a harsher sentence if the perpetrator of the femicide is a man.
7. Legal sanctions for femicide differ radically across the six countries, ranging from 15 years of imprisonment to life. However, there is no relation between the length of the sentences foreseen in the CC and the number of femicides in each country. This corroborates the notion that imposing the harshest possible sanctions, such as life imprisonment, does not guarantee any deterrent effect. This is a result that refutes the claims made by activists who have been promoting and imposing harsher laws as the solution to reduce femicides.
8. The results of this research challenge explanations of femicide based on routine activities theory. In these kinds of explanations, the missing element appears to be a punctual event that motivates the murderer to take action. On the basis of the research available, that event could well be the perpetrators’ awareness of the fact that their relationship is over and their partners are moving on with their lives. However, the vast majority of abandoned partners do not aggress against their partners, which shows the limitations of the current explanations of femicide.
9. This research contributes to a growing literature which shows that criminologists have not found a scientific explanation of femicide yet, leaving the field open for the promulgation of laws guided by ideology instead of evidence-based research.
10. To improve research on femicide and develop efficient prevention programs, we suggest setting aside research models based on the study of known cases of femicide to establish the profile of the murderers. Instead, we recommend placing the study of femicides in the general context of homicides and crimes against persons to understand their similarities and differences better.
11. Finally, knowing that human aggression is the result of the combination of inherited and environmental influences on human behavior, we propose a holistic approach that incorporates biology, neurosciences, and psychology, as well as alternative sociological and cultural explanations.

## Notes

1. *The New York Times* ran a similar article on April 6, 2020 (updated on April 14) stating that “As quarantines take effect around the world, that kind of intimate terrorism—a term many experts prefer for domestic violence—is flourishing” and argued that the number of cases reported to the authorities or the telephone calls to helplines were on the rise in China, France, Italy, Spain, and the United Kingdom (Taub, 2020).

2. As suggested by Gonzalez et al. (2020: 1), the corroboration of this kind of hypothesis within the framework of the pandemic could have “important implications for policy and the virus mitigation efforts, which might urge policymakers to terminate stay-at-home orders in an effort to reduce family violence and other social risk factors.” The consequences would be far worse if the research findings were inaccurate because lifting the lockdowns prematurely “may ultimately result in more COVID-related deaths” (Gonzalez et al., 2020: 1).

3. According to this view, routine activity theory suits better crimes between strangers than those among kin (in that sense, see Miró-Llinares, 2014).

4. In contrast, criminologists like Piquero et al. (2020) applied routine activities theory to predict increases in domestic violence cases as a consequence of the lockdowns, which corresponds to the situational hypothesis presented above.

5. A review of the research findings from different fields concluded that the measures taken to control the virus during 2020 had adverse effects on the population’s economy and mental health, including increases in depression, anxiety, alcohol, and drug intake, and, as criminologists predicted, domestic conflicts (Cohut, 2021).

6. We are aware that it would be better to estimate the rate per 100,000 women inhabitants of the country, but the number of women in the total population was not available for all of the countries studied.

7. https://www.argentina.gob.ar/generos/linea-144/informacion-estadistica.

8. Weil (2020) qualified femicide as another “global pandemic” and indicated that the figures available for some countries, including Argentina and Spain, at the beginning of the lockdowns were surprisingly high; nevertheless, the author did not standardize the data according to the population of each country and did not have a point of comparison with former years.

9. This profile can be observed in Schaller’s (2021) research, who analyzed all of the IPH recorded in one Swiss canton during several years, and found that there is often a specific event—in general, the perpetrator’s realization that the romantic relationship is definitively over—that serves as a catalyst of the femicide, independent of the offender’s previous record.

10. From a statistical point of view, the number of cases recorded in Panama is too small to allow valid conclusions about their seasonal distribution to be drawn.

11. See the data available at https://www.observatoriofeminicidioscolombia.org.

12. If we enter the field of conjectures, one can also speculate, as an anonymous reviewer suggested, that an external threat usually strengthens the ties between relatives, independent of their differences. From that perspective, the threat of death by COVID-19 could have even served as a protective factor against domestic aggression. We recognize that this kind of reaction has been observed at the macro-level when, for example, a country goes to war: both Margaret Thatcher and George Bush, Jr. won elections after entering the Falkland/Malvinas war and the second Iraqi war, respectively, although their popularity was at an all-time low before them. However, we do not have data to test such a hypothesis properly, and above all, we have seen that research on trends in domestic violence overall showed that it increased during the lockdowns.

13. Felson (1995) has recognized that “[o]riginally the routine activity approach took offenders as given” but that later approach was linked to control theory (Hirschi, 1969) to take “into account social control of offenders” (Felson, 1995, p. 54). From that perspective, one can say that both theories share as a postulate that humans have a general predisposition toward deviance that would take the lead if social bonds are weak (in Hirschi’s control theory), or in the absence of a handler who could supervise the likely offender when confronted with a suitable target (in Felson’s revised version of the routine activities approach). However, neither of these was developed to explain femicides. In particular, control theory (Hirschi, 1969) is mainly a theory of juvenile delinquency.

14. Studies of violent offenders’ brains based on neuroscientific methods are published seldom in criminology journals, even when they make reference to criminological theories (Carlisi et al., 2020). Similarly, psychopathology is included rarely in criminological studies of intimate partner violence, although one can find a noteworthy exception in Cunha et al.’s study (2021).

15. Sapolsky (2017) mentioned Elias’s theory only once and indirectly through Pinker’s study (2011).

16. See the dedication in later editions of *The Civilizing Process* (Elias, 1939/2000).

17. Norbert Elias, as Auguste Comte before him and Robert Sapolsky and Steven Pinker today, can be seen as examples of the way interdisciplinary research can be conducted in practice.

18. On September 27, 2021, when the final version of this article had just been accepted for publication, the Federal Bureau of Investigation of the United States of America published its annual police recorded crime statistics, which also showed a decrease in domestic violence murders in 2020. *The New York Times* covered this information through a long article written by Pulitzer award winner Neil MacFarquhar (MacFarquhar, 2021), which focuses on the rise of the total number of murders. “And while domestic violence killings dropped slightly from recent years, they were still a factor” writes MacFarquhar before engaging himself in a detailed description of one of these murders. The readers get to know the name of the perpetrator, the smallest details of the garment he used to strangle his victim (“a sleeveless white T-shirt”), and learn that he did not kill her because she was a woman, but because he “became convinced that she was a demon who could hurt her family.” The description is so vivid that one wonders whether the readers will keep that murder in mind instead of the positive news of a decrease in IPH. This is particularly striking as the decrease of that subcategory of murders was unattended in the context of the coronavirus-related lockdowns, in the framework of a rise by almost 30% of the total number of murders in the United States in 2020, and it contradicts the predictions made by the same journal at the beginning of the stay-at-home restrictions (see footnote 1). As noted by Pinker (2018), this preference of the media for the negative news has major consequences on the population because it distorts people’s view of the world, inducing pessimism and “a sense of gloom about the state of the world” (Pinker, 2018: 42).
